# Clinical Course and Treatment Implications of Combination Immune Checkpoint Inhibitor-Mediated Hepatitis: A Multicentre Cohort

**DOI:** 10.1093/jcag/gwab019

**Published:** 2021-07-28

**Authors:** Matthew K Smith, Yin Chan, Aleksi E Suo, Abdel Aziz Shaheen, Stephen E Congly, Puneeta Tandon, Rahima A Bhanji, Malcolm M Wells, Tina Cheng, Christopher Ma

**Affiliations:** 1 Department of Medicine, University of Alberta, Edmonton, Alberta, Canada; 2 Division of Gastroenterology and Hepatology, University of Calgary, Calgary, Alberta, Canada; 3 British Columbia Cancer Agency, University of British Columbia, Abbotsford, British Columbia, Canada; 4 Department of Community Health Sciences, Cumming School of Medicine, University of Calgary, Calgary, Alberta, Canada; 5 O’Brien Institute for Public Health, University of Calgary, Calgary, Alberta, Canada; 6 Division of Gastroenterology and Hepatology (Liver Unit), University of Alberta, Edmonton, Alberta, Canada; 7 Division of Medical Oncology, Department of Oncology, University of Calgary, Calgary, Alberta, Canada

**Keywords:** Hepatitis, Immune checkpoint inhibitor, Immune-related adverse event, Ipilimumab, Nivolumab

## Abstract

**Background:**

Immune-related adverse events can occur after treatment with immune checkpoint inhibitors (ICI), limiting treatment persistence. We aimed to evaluate the clinical course of ICI-mediated hepatitis (IMH) associated with combination ipilimumab and nivolumab treatment.

**Methods:**

A retrospective cohort study including consecutive patients with metastatic melanoma treated with ipilimumab and nivolumab between 2013 and 2018 was conducted at two tertiary care centres. IMH was defined by the Common Terminology Criteria for Adverse Events (CTCAE). We determined the proportion of patients developing IMH, and compared the duration, treatment patterns and outcomes, stratified by hepatitis severity. Kaplan–Meier survival analysis was used to evaluate time to hepatitis resolution, and a linear mixed-effects model was used to compare longitudinal outcomes by treatment.

**Results:**

A total of 63 patients were included. Thirty-two patients (51%) developed IMH (34% Grade 1–2, 66% Grade 3–4), at a median of 34 days (IQR 20 to 43.5 days) after the first dose. Baseline FIB4 index ≥1.45 was associated with IMH (OR 3.71 [95% CI: 1.03 to 13.38], *P* = 0.04). Ninety-four per cent (30/32) of patients had liver enzyme normalization after a median duration of 43 days (IQR 26 to 70 days). Corticosteroid use was not associated with faster IMH resolution or less ICI discontinuation. A total of 24 patients died during the study; no deaths were attributable to hepatitis-related complications. Fifty-three per cent (17/32) of patients resumed anti-PD-1 monotherapy and three patients developed IMH recurrence.

**Conclusions:**

Approximately half of the patients treated with combination ipilimumab and nivolumab developed IMH in this cohort. However, most patients experienced uncomplicated IMH resolution.

## Introduction

The immune system plays an integral role in cancer recognition and tumour control (1,2). Consequently, the advent of immunotherapies that stimulate an anti-neoplastic adaptive T-lymphocyte response has revolutionized the modern management of several advanced cancers (3–9). In patients with advanced melanoma, treatment with immune checkpoint inhibitor (ICI) monoclonal antibodies targeting programmed cell death protein 1 (PD-1) (e.g., nivolumab) either alone or in combination with anti-cytotoxic T-lymphocyte-associated protein 4 (CTLA-4) (e.g., ipilimumab) improves survival outcomes and are now standard-of-care treatment options (3–5).

Combination ICI regimens may have more potent anti-neoplastic effects in some patients, but are also associated with a greater degree of treatment-related morbidity from immune-related adverse events (irAEs) (10). The immune checkpoint pathways are important regulators of self-tolerance. Therefore, treatment with ICIs can result in irAEs affecting nearly all organ systems (11). In a meta-analysis of randomized controlled trials, Da et al. demonstrated that approximately 1 in 10 patients treated with ICI monotherapy developed immune-mediated hepatitis (IMH) and combination ICI treatment was associated with a nearly threefold increased risk of severe disease (12). Other retrospective cohort studies have demonstrated incidences of IMH as high as 20% (13,14).

The development of irAEs carries therapeutic implications as severe or recurrent irAEs can be life threatening and may limit continuation of ICI treatment. While the combination of ICIs is known to increase the risk of IMH, specific patient- or disease-related factors that are predictive of IMH have not yet been identified. Similarly, there are no known predictors of IMH severity or disease course. Current guidelines from the Society for Immunotherapy of Cancer Toxicity Management Working Group and American Society of Clinical Oncology recommend treatment of IMH with immunosuppressive therapy such as corticosteroids alongside withholding immunotherapy in patients with grade 2 or more severe disease (15,16). However, it is unclear if all patients require these interventions.

Therefore, we aimed to characterize the clinical course, treatment, and outcomes of patients developing IMH in a retrospective, multicentre cohort of patients treated with combination nivolumab and ipilimumab for advanced or metastatic melanoma.

## MATERIALS AND METHODS

### Study Design, Setting, and Data Source

This retrospective cohort study was conducted using data collected from consecutive patients with unresectable stage 3 or 4 melanoma treated with combination ipilimumab and nivolumab at the Tom Baker Cancer Centre, Calgary, Canada and Cross Cancer Institute, Edmonton, Canada, between 2013 and 2018. Patient data from both centres were collected from a shared, comprehensive provincial electronic health record, which contains information on outpatient and inpatient visits, laboratory data, diagnostic imaging and pharmaceutical prescriptions.

### Patient Population

Our study cohort was identified from a clinical database of melanoma patients from the Tom Baker Cancer Centre, Calgary and Cross Cancer Institute, Edmonton. Demographics, disease staging and treatment information was registered by the patient’s primary oncologist and corroborated using the provincial cancer electronic medical record, which contains data on all prescription medication use including anti-neoplastic therapies. Eligible patients with advanced melanoma received induction nivolumab 1 mg/kg and ipilimumab 3 mg/kg administered intravenously (IV) once every 3 weeks for up to four cycles followed by nivolumab 1 mg/kg IV every 2 weeks maintenance therapy. All patients who received at least one dose of combination treatment were included. Patients with cirrhosis, chronic infection with hepatitis B or C, or known co-existing liver diseases were excluded. Monitoring of bloodwork, including alanine transferase (ALT), aspartate aminotransferase (AST), alkaline phosphatase (ALP) and total bilirubin were performed prior to each treatment per protocol. Measurements of the international normalized ratio (INR) and albumin were performed at the treating physician’s discretion. All patients had cross-sectional imaging for disease staging prior to the initiation of treatment.

### Outcomes and Definitions

The primary outcome of interest was the development of IMH, as per the treating physician’s judgment, while receiving the first four cycles of induction combination nivolumab with ipilimumab treatment, graded using the Common Terminology Criteria for Adverse Events (CTCAE) version 5.0. Subsequently, detailed medical chart review for all ICI-related adverse events was conducted (including IMH) and verified. The CTCAE grade was calculated for each patient using their corresponding laboratory values. Grade 1–2 hepatitis was defined by an asymptomatic rise in the AST or ALT ≤5× the upper limit of normal (ULN), with a Roussel Uclaf Causality Assessment Method (RUCAM) score ≥6, indicating probable drug-induced liver injury (17). Grade 3 hepatitis was defined by symptomatic liver dysfunction, AST or ALT 5-20× ULN, or bilirubin 3-10× ULN. Grade 4 hepatitis was defined by decompensated liver function, AST or ALT > 20× ULN, or total bilirubin >10× ULN. The ULN was defined using sex-appropriate cut-offs (males: ALT 33 U/L, AST 40 U/L, ALP 115 U/L; females: ALT 25 U/L, AST 32 U/L, ALP 100 U/L). Baseline liver enzymes were defined as the liver enzyme profile of the patient within one month prior to initiating combination ICI treatment. For patients with abnormal baseline liver enzymes, these definitions were adapted to reflect change relative to the baseline rather than the ULN. During the study period, liver biopsies were not routinely performed in our centres after the initiation of combination ICI therapy to assess for disease-specific histopathology. For patient FIB4 index, FIB4 index = (patient age [years] × AST [U/L])/(Platelet Count ([0^9^/L] × √ALT [U/L]). Transient elastography was not performed in this cohort.

Secondary outcomes of interest included time to development of IMH and time to peak liver enzyme elevation (from the first dose of combination ICI treatment), total hepatitis duration (defined from the time of onset to time of liver chemistry normalization), post-hepatitis ICI treatment and death. Treatment of IMH and non-chemotherapeutic drug use was collected from each patient’s electronic medical record: sources searched for medication use included physician notes for treatment, histories and discharge summaries, and the patient’s medication profile. IMH treatment was categorized as: 1) no treatment; 2) temporary or permanent combination ICI discontinuation; 3) corticosteroid use and 4) biologic treatment. Corticosteroid use was further stratified by route of administration (intravenous versus oral). Corticosteroid dose was decided by the treating physician. Other variables of interest included patient age, sex, cancer staging, previous anti-neoplastic therapy, concurrent liver disease (including radiographic evidence of hepatic steatosis by radiologist report from computed tomography or ultrasound assessment within 12 months of combination ICI use, or suspected liver metastases), baseline Fibrosis-4 [FIB4] index and development of other irAEs (including ICI-related enterocolitis or diarrhoea) as documented by the treating physician.

### Statistical Methods

Descriptive statistics for the cohort are presented as medians with interquartile range for nonparametrically distributed continuous variables, and proportions for categorical variables. Differences in baseline characteristics between patients developing IMH and patients without IMH were evaluated using the Mann–Whitney *U* test for distribution of nonparametric continuous variables, or Pearson Chi-squared test for proportions. Differences in paired continuous data between baseline, onset, peak and resolution of hepatitis were evaluated using the Wilcoxon signed-rank test. To evaluate time to onset of hepatitis, time to peak liver enzyme elevation and time to hepatitis resolution, lifetables were constructed and analysed using the Kaplan–Meier method. Differences between IMH CTCAE grade groups were evaluated using the log rank test. Changes in the relative value of liver enzymes to the sex appropriate ULN from baseline to onset of hepatitis, peak liver enzyme elevation, and after resolution are depicted graphically in violin plots. Violin plots show the median, interquartile range and probability density of data at different values, smoothed using a kernel density estimator. A univariable logistic regression was used to identify associations between baseline factors and development of IMH, expressed as unadjusted odds ratios (OR) with 95% confidence intervals (CI). Multivariable modelling was not performed due to the small sample size.

All analyses were conducted in STATA 16.1 (StataCorp LLC, College Station, TX). The Health Research Ethics Board of Alberta, Cancer Committee approved the study with patient informed consent waived (CC-17–0435).

## RESULTS

### Patient Demographic Characteristics

A total of 63 patients with malignant melanoma treated with combination ipilimumab-nivolumab were included in the cohort. Baseline demographic characteristics stratified by development of IMH are summarized in Table 1. All patients were started on therapy for advanced melanoma (92% for stage IV, 8% for unresectable stage III). A total of 33 patients (52%) were BRAF mutation positive, although only 10 patients received dabrafenib and/or trametinib as first-line agents. No patients received either dabrafenib or trametinib within 30 days prior to starting combination ICI. Eight patients (13%) had uveal melanoma, 55 (87%) had cutaneous melanoma. Median age at time of combination ICI therapy was 55 years (IQR 44 to 61 years). A total of 28 patients (44%) underwent four complete cycles of combination ICI therapy, whereas 6 patients (10%) only completed a single cycle of treatment. Viral hepatitis serology was re-assessed in 37 of 63 patients, with no patients identified as being positive. No patients were identified as having significant alcohol use disorder. Radiographic evidence of hepatic steatosis was demonstrated in 13% (8/63) of patients and 41% (26/63) of patients had evidence of hepatic metastases. Concurrent irAEs were present in 34/63 patients (54%), including immune-related dermatitis, hypophysitis, myositis and nephritis. Checkpoint inhibitor associated enterocolitis developed in 26 patients (41%).

**Table 1.** Baseline demographic characteristics of patients treated with combination ipilimumab and nivolumab for metastatic melanoma (2013–2018)

Characteristic	No ICI-related hepatitis (*n* = 31)	ICI-related hepatitis (*n* = 32)	*P*-value
Median age (years, IQR)	55 (43, 61)	53.8 (13.8)	0.66
Male sex (*n*, %)	19 (61%)	22 (69%)	0.53
Median cycles of ICI treatment (*n*, IQR)	3 (2, 4)	3 (2, 4)	0.24
Other immune-related AE (*n*, %)	18 (58%)	16 (50%)	0.52
ICI-related enterocolitis/diarrhoea	15 (48%)	11 (34%)	0.26
Immune checkpoint inhibitor hepatitis grade* (*n*, %)			
CTCAE Grade 1–2	-	11 (34%)	-
CTCAE Grade 3–4		21 (66%)	
Pre-existing liver disease (*n*, %)			
Hepatic steatosis	2 (6%)	6 (19%)	0.14
Liver metastases at treatment	12 (39%)	14 (44%)	0.68
Median baseline liver tests (IQR)			
AST (U/L)	26 (20, 29)	30 (23, 34)	0.05
ALT (U/L)	31 (23, 37)	32.5 (26.5, 38)	0.31
ALP (U/L)	95 (61, 116)	76 (61.5, 97.5)	0.13
Total Bilirubin (µmol/L)	8 (6, 11)	10.5 (6.5, 12)	0.24
Baseline Fibrosis-4			
<1.45	27 (87%)	20 (65%)	0.04
≥1.45	4 (13%)	11 (35%)	
Median BMI (IQR)	28.6 (26.3, 34.2)	27.8 (25.3, 31.6)	0.26

ALP, Alkaline phosphatase; ALT, Alanine transaminase; AST, Aspartate aminotransferase; CTCAE, Common Terminology Criteria for Adverse Events; ICI, immune checkpoint inhibitor; IQR, Interquartile range; ULN, Upper limit normal.

*Hepatitis grading based on Common Terminology Criteria for Adverse Events; grade 1–2 (AST or ALT >3–5× ULN, ALP >2.5–5× ULN, bilirubin >1.5–3× ULN), grade 3 (AST or ALT >5–20× ULN, ALP >5–20× ULN, bilirubin >3–10× ULN), grade 4 (AST or ALT >20× ULN, ALP >20× ULN, bilirubin >10× ULN).

### Incidence and Severity of IMH

A total of 32 patients (51%) developed hepatitis after receiving ipilimumab and nivolumab: 11/32 patients (34%) developed grade 1–2 hepatitis and 21/32 patients (66%) developed grade 3–4 hepatitis. No patients developed fulminant liver failure. Among patients who did not have hepatic metastases, 49% (18/37) developed IMH after combination ICI treatment. Patients who developed IMH had a higher baseline median AST (30 U/L versus 26 U/L, *P* = 0.05) compared to patients who did not develop IMH and trended toward having a higher median FIB4 (1.22 versus 1.00, *P* = 0.09). Baseline ALT, ALP and bilirubin were similar.

**Figure 1.**
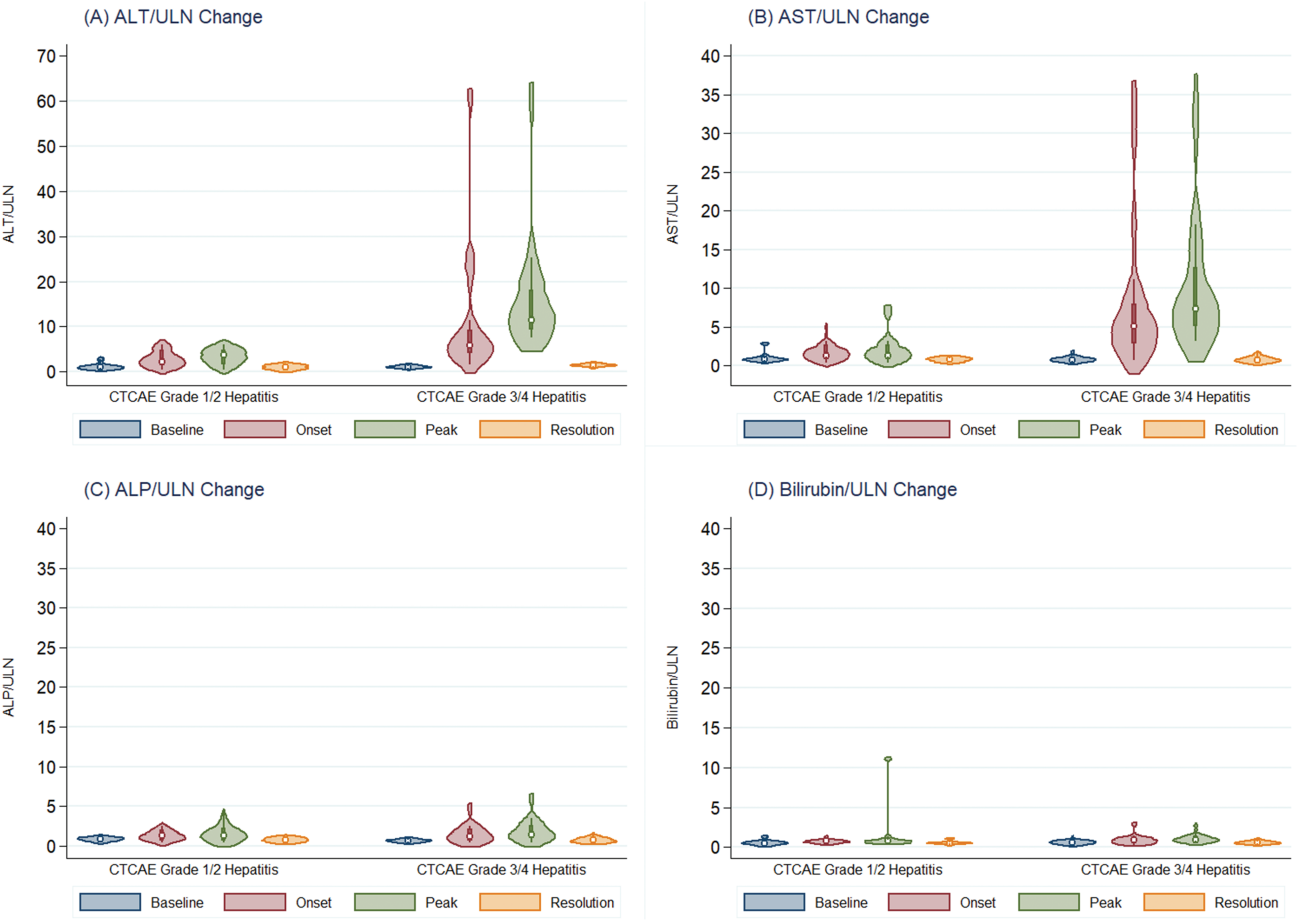
Violin plots demonstrating the distribution of alanine aminotransferase (A), aspartate transaminase (B), alkaline phosphatase (C) and total bilirubin (D) from baseline (blue) to onset of ICI-mediated hepatitis (red), peak hepatic inflammation (green) and hepatitis resolution (yellow). White circles represent median, boxes represent interquartile range, width of violin plots represent probability density of data at each value relative to the upper limit of normal.

**Figure 2.**
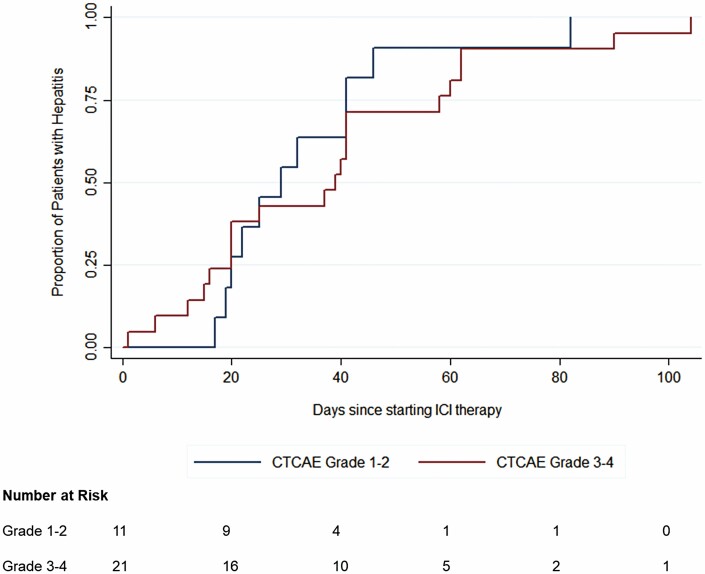
Kaplan–Meier failure curve for onset of immune checkpoint inhibitor-mediated hepatitis, stratified by CTCAE grade. No significant difference in onset between grade 1–2 hepatitis vs. grade 3–4 hepatitis (log rank *P*-value = 0.51).

The median change in liver enzymes and bilirubin from baseline to the onset of hepatitis, peak enzyme elevation and post-ICI-hepatitis resolution are summarized in Figure 1. Most patients had a hepatocellular-predominant pattern of liver enzyme elevation, with only three patients (9%) developing cholestatic-predominant patterns. Between baseline and onset of hepatitis, there were significant differences in the median ALT:ULN ratio (4.9 versus 1.0, *P* < 0.0001), AST:ULN ratio (3.1 versus 0.8, *P* < 0.0001), ALP:ULN ratio (1.2 versus 0.7, *P* < 0.0001) and bilirubin:ULN ratio (0.8 versus 0.6, *P* = 0.005). No patients had an isolated ALP elevation. Median time to hepatitis onset was 34 days (IQR 20, 43.5) after first combination ICI dose (Figure 2). Median time to peak enzyme elevation was 48 days (IQR 23.75, 76.5) from onset of hepatitis. There was no significant difference in onset between patients with grade 1–2 versus grade 3–4 hepatitis (log-rank *P*-value = 0.51), nor were there any differences by baseline FIB4 (<versus≥1.45, *P* = 0.14), sex (*P* = 0.90), hepatic metastases (*P* = 0.26), hepatic steatosis (*P* = 0.08) or other concomitant irAEs (*P* = 0.62) (Supplementary Figure 1). A total of 12 patients (38%) developed an elevated bilirubin ≥20 µmol/L; none had an associated increase in INR/PTT or developed acute liver failure.

On univariable analysis, an elevated baseline FIB4 index ≥1.45 was significantly associated with the development of IMH (OR 3.71 [95% CI: 1.03 to 13.38], *P* = 0.04). Patients with an elevated baseline AST (OR 1.27 per 5 U/L elevation [95% CI: 0.97 to 1.69], *P* = 0.08) trended toward having a higher risk of IMH although this was not statistically significant. Male sex, presence of steatosis, presence of liver metastases at baseline, development of other immune-related adverse events and baseline ALT, ALP or bilirubin were not significantly associated with IMH.

### Treatment of IMH

Corticosteroid treatment was initiated in 97% (31/32) of patients who developed IMH during the study period. Five patients (16%) were admitted as inpatients for treatment of IMH. There were four patients (13%) who developed IMH while already on corticosteroid therapy. The mean induction corticosteroid dose was 69 mg (± 23 mg) prednisone-equivalent/day (adjusted for weight, mean dose of 0.86 mg/kg (± 0.21 mg/kg). Intravenous corticosteroids were used in five patients (16%) at induction. One patient required a single dose of intravenous 5 mg/kg infliximab for management of IMH that was unresponsive to corticosteroids. Of note, no patients within our cohort received MMF or cyclosporine. Combination ICI treatment was discontinued in 62.5% (20/32) of patients who developed IMH.

### IMH Resolution

**Figure 3.**
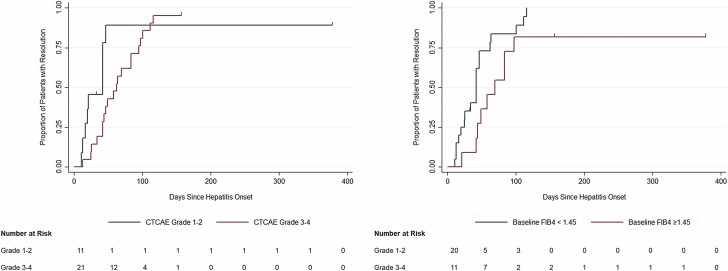
Kaplan–Meier survival curve for resolution of immune checkpoint inhibitor-mediated hepatitis. No significant difference in resolution by hepatitis grade (Figure 3A) (log rank *P*-value 0.12), by baseline FIB4 index (Figure 3B) (log rank *P*-value 0.05).

Of the 32 patients who developed IMH, 30/32 (94%) had eventual resolution and normalization of liver enzymes (Figure 1). The median time to hepatitis resolution was 43 days (IQR 26, 70), with a trend toward faster resolution in patients with grade 1–2 hepatitis compared to patients with grade 3–4 hepatitis (27 versus 60 days, *P* = 0.12), and a trend toward faster resolution among patients with a baseline FIB4 index <1.45 (42 versus 58 days, *P* = 0.05) (Figure 3A,B). Higher induction corticosteroid dosing was not significantly associated with faster resolution of IMH (HR 0.90 [95% CI: 0.74 to 1.10], *P* = 0.31 per 0.1 mg/kg/day increase in dose). Compared to the baseline, there were no significant difference in post-hepatitis resolution AST:ULN ratio (0.7 versus 0.8, *P* = 0.67), ALP:ULN ratio (0.7 versus 0.7, *P* = 0.36) or bilirubin:ULN ratio (0.6 versus 0.5, *P* = 0.47). There was a trend toward a higher ALT:ULN ratio post-hepatitis resolution compared to baseline (1.4 versus 1.0) although this did not reach statistical significance (*P* = 0.06).

### Other Outcomes

Among the 32 patients who developed IMH, 17 patients (53%) restarted post-combination therapy with PD1 inhibitor monotherapy. Three patients (18%) restarting a PD1 inhibitor re-developed IMH (two patients with Grade 3 IMH, one patient with Grade 2 IMH). A total of 24 patients (38%) died in the cohort, most commonly from sequalae of their progressive metastatic disease manifesting as multi-organ dysfunction. Cause of death was obtained from the last physician documentation in the death summary from the patient’s electronic medical record; no deaths directly attributable to hepatitis were identified.

## Discussion

Understanding the clinical course of ICI-mediated adverse events, such as IMH, is particularly relevant as combination immunotherapy becomes the standard of care, despite a greater risk of immune-related complications. In this multicentre retrospective cohort study of 63 patients with unresectable melanoma treated with combination ipilimumab and nivolumab, we found that approximately half of patients developed significantly elevated liver enzymes likely secondary to IMH, with no patients developing fulminant liver failure. Almost all patients had complete normalization of liver enzymes over time, with no significant differences in hepatitis outcomes demonstrated between IMH grade or with higher dose corticosteroid exposure.

In a recent systematic review, Peeraphatdit et al. determined that the incidence of IMH in patients undergoing ICI treatment was highly variable, ranging from 0.7% to 16% depending on class of therapy and therapeutic dose (18). In contrast, a higher proportion of patients developed liver injury after ICI exposure in our study, in keeping with data from the French CERTIM group (Immunomodulatory Therapies Multidisciplinary Study group), who showed that 23% of patients exposed to any ICI therapy developed grade 3–4 IMH (19). The authors hypothesized that both selection and reporting bias favouring severe cases of hepatitis was associated with differences in the reported incidence. Furthermore, IMH remains a clinical diagnosis of exclusion, and is dependent on the evaluation for other causes of hepatotoxicity. A diagnosis of exclusion can be particularly challenging in the neoplastic setting if there are concurrent infiltrating liver metastases (which may undergo necrosis with ICI treatment) or other immune-related adverse events such as myositis or myocarditis with secondary transaminitis (20–22). However, we excluded patients with liver metastases in a sensitivity analysis, and this did not appreciably change our point estimates. While we applied the RUCAM score to identify patients with probable to highly probable IMH, the discriminatory capacity of RUCAM for IMH requires validation because the hypothesized injury mechanism is related to indirect hepatotoxicity from immune activation, rather than conventional drug-induced liver injury from direct or idiosyncratic hepatotoxicity (23).

The lack of a diagnostic gold standard for IMH has limited the development of consensus diagnostic criteria or predictors based on biologic or histopathologic features (24,25). In our study, higher baseline FIB4 index was associated with a greater risk of IMH, although we were unable to adjust for other confounders in multivariable logistic regression analysis due to inadequate power. While FIB4 has only been validated in the study of chronic liver disease, we hypothesize that higher baseline FIB4 index may reflect a degree of underlying liver disease that was not identified pre-treatment, as no patients in our study underwent a liver biopsy as part of routine care pre-treatment. Radiographic hepatic steatosis, presence of hepatic metastases at baseline (26), and development of other irAEs or ICI-associated enterocolitis were not significantly associated with IMH development, although we are limited by sample size. Currently, no biomarkers or clinical features reliably predict IMH. Potential candidate biomarkers include interleukin (IL)-6 (27), soluble CD163 and CXCL5 (28), and IL17 (29). However, these require independent validation prior to integration into routine clinical practice.

Almost all patients in our cohort were treated with corticosteroids and most patients had ICIs temporarily or permanently withheld in accordance with current clinical practice guidelines (15,16,30). As previously described, most cases of IMH appear to resolve independent of steroid dose and the efficacy of high dose corticosteroids for other causes of drug-induced liver injury remains contentious (31). Corticosteroid exposure is also associated with a sevenfold increased risk of infections in this population and there is a theoretical risk that immunosuppression may decrease the efficacy of ICIs (32,33). In our study, similar to Cheung et al., we did not demonstrate a significant benefit with higher dose corticosteroid exposure with respect to the continuation of combination ICI therapy, resolution or time to improvement of IMH (13). While we recognize the necessity for treatment in patients with CTCAE grade 3 and 4 IMH to avoid fulminant hepatitis and subsequent complications, the current recommendations for corticosteroid dosing in IMH requires evaluation in a prospective trial.

For patients who have IMH resolution after discontinuation or completion of combination ICI treatment, the benefits of restarting ICI monotherapy should be considered with the potential risk of irAE reactivation. In our cohort, 14/17 patients were able to safely restart anti-PD1 monotherapy after IMH, which mirrors the positive re-exposure experience reported by other authors (34). Reassuringly, we did not observe any cases of drug-induced acute liver failure in our cohort, in keeping with other studies demonstrating a 0.1% to 0.2% risk of ICI-induced fulminant hepatic failure.

Our study has several important limitations. First, our study was conducted retrospectively, and the inherent limitations of this study design should be considered, including the risk of recall and selection bias. Second, there are limitations around diagnostic definitions of IMH that have been previously discussed. As this is a diagnosis of exclusion, it is particularly difficult to discern retrospectively. We excluded patients with grade 1–2 hepatitis with low RUCAM scores and performed a sensitivity analysis excluding patients with hepatic metastases. However, some degree of residual diagnostic uncertainty remains. Third, despite combining data at two tertiary care sites, our sample size was relatively small. Fourth, no patients in our cohort underwent a liver biopsy so we are unable to correlate clinical outcomes with histologic patterns of disease, preventing the ability to perform subgroup analyses stratified to histopathologic subtype. Finally, the treatment pattern (withholding ICIs and administration of corticosteroids) was similar for almost all patients, so we were unable to specifically evaluate the efficacy of different management strategies. However, we did apply different methods to evaluate the effect of high versus low-dose corticosteroid exposure, which is an important clinical question in this population.

In summary, we describe a multicentre cohort of patients with advanced melanoma treated with combination ipilimumab and nivolumab, with approximately half of patients developing IMH within 1 to 2 months of the first treatment cycle. We identified that IMH development was associated with a higher baseline FIB4 index, although other demographic and biochemical parameters were not predictive. Almost all patients in our cohort experienced IMH resolution with no persistent hepatic dysfunction, irrespective of corticosteroid dosing. Prospective controlled studies are needed to elucidate the precise role of immunosuppression in patients developing IMH.

## SUPPLEMENTARY DATA

Supplementary data are available at *Journal of the Canadian Association of Gastroenterology* online.

Supplemental Figure 1. Onset of immune checkpoint inhibitor-associated hepatitis by sex, existing liver disease, and concomitant immune-related adverse events.

gwab019_suppl_Supplementary_Figure_1

## Funding

None declared.

## Authors contribution

M.S.: study design and conception, data collection, manuscript drafting and editing for important intellectual content. Y.C.: study design and conception, data collection, manuscript editing for important intellectual content. A.E.S.: study design and conception, data collection, manuscript editing for important intellectual content. A.A.S.: manuscript editing for important intellectual content. S.E.C.: manuscript editing for important intellectual content. P.T.: manuscript editing for important intellectual content. R.A.B.: manuscript editing for important intellectual content. M.M.W.: manuscript editing for important intellectual content. T.C.: study design and conception, manuscript editing for important intellectual content. C.M.: study design and conception, manuscript drafting and editing for important intellectual content. All authors have contributed and approved the final version of this manuscript. C.M. is acting as the article guarantor.

## Conflicts of interest

A.A.S.: Grants from Gilead Sciences and Intercept Pharmaceuticals. S.E.C.: Grants from Gilead Sciences, Boehringer Ingelheim, Genfit, Allergan, Sequana Medical, and personal fees from Intercept Pharmaceuticals and Eisai outside the submitted work. The other authors have no conflicts of interest to declare.
