# Supplementary material for: Clinical Course and Treatment Implications of Combination Immune Checkpoint Inhibitor-Mediated Hepatitis: A Multicentre Cohort
Source: J Can Assoc Gastroenterol. 2021 Jul 28;5(1):39–47. doi: 10.1093/jcag/gwab019 (PMC8806044; doi:10.1093/jcag/gwab019)
Supplement: gwab019_suppl_Supplementary_Figure_1 [file gwab019_suppl_supplementary_figure_1.docx]

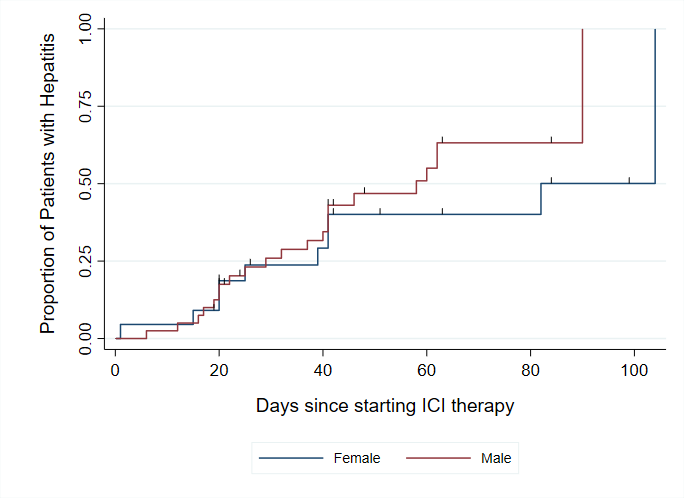

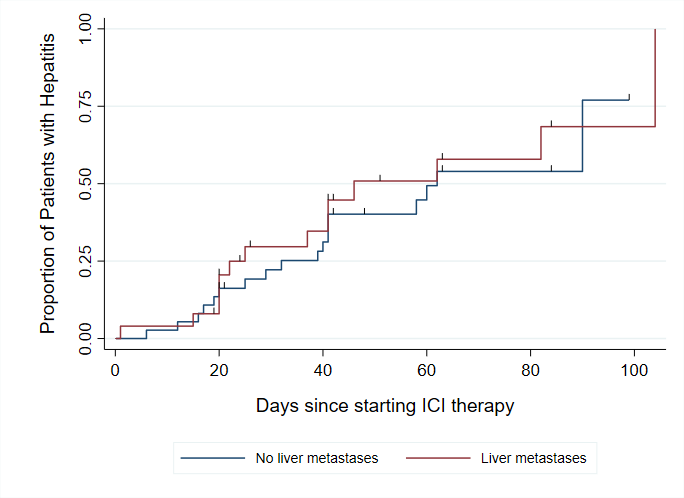

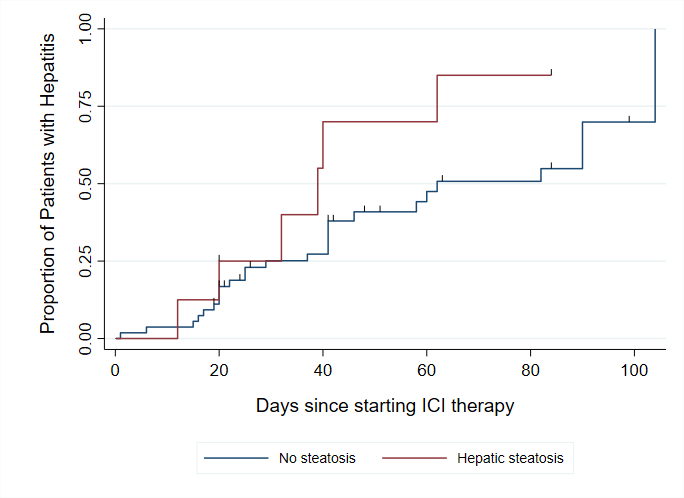

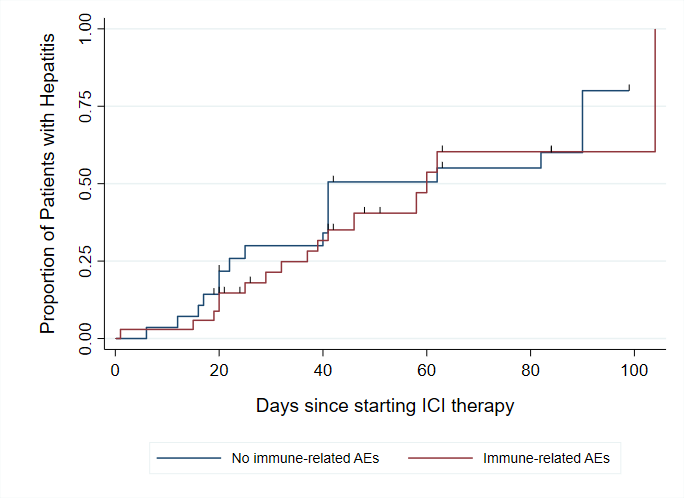


**Supplemental Figure 1.** Onset of immune checkpoint inhibitor-mediated colitis by sex, existing liver disease, and concomitant immune-related adverse events
